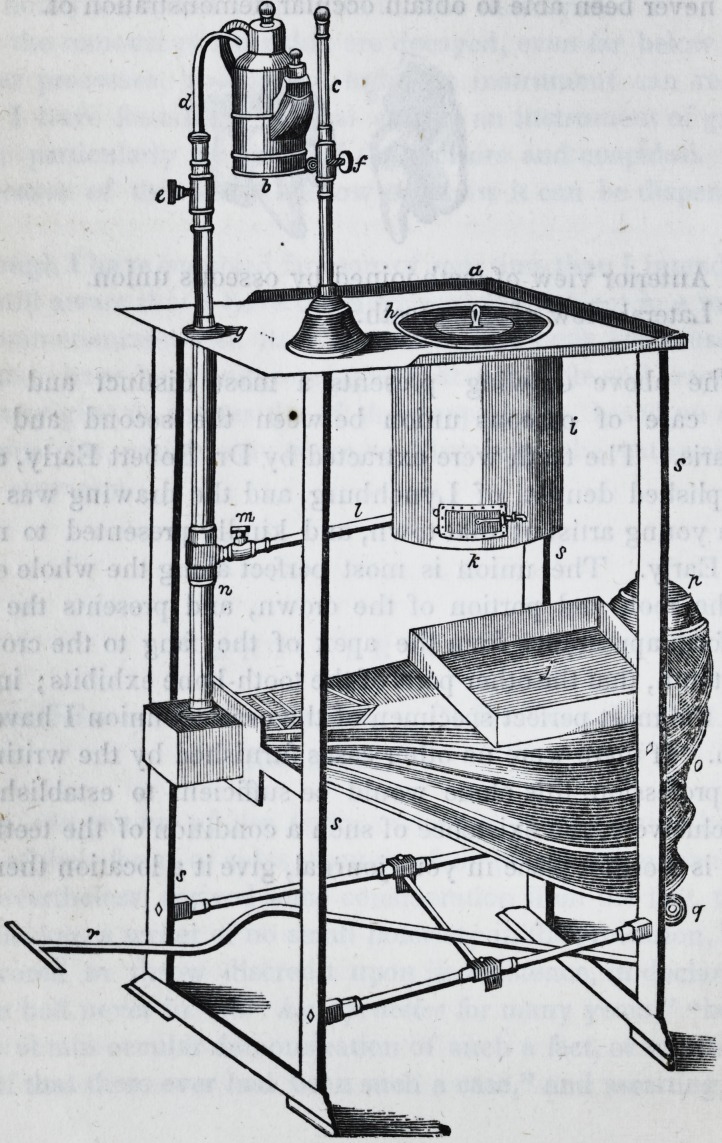# Dr. Somerby's Patent Blow-Pipe and Furnace

**Published:** 1845-03

**Authors:** R. Somerby


### ARTICLE VII.

### *Dr. Somerby's Patent Blow-pipe and Furnace.*

DR. SOMERBY having furnished us with the following engraving and description of his patent blow-pipe and furnace, we insert for the benefit of our readers.—EDS.

This instrument, I designed especially for dentists, but it is capable of being applied to many kinds of business. To the chemist, mineralogist, and for various experiments in chemical laboratories, and colleges, and for every thing requiring a steady heat from the blow-pipe, or furnace, this is one of the most valuable machines ever offered to the public. More soldering can be done by it in an hour, than can be performed by the most skilful artist in ten, where the blast is produced by the mouth. The largest piece of work that is put up by the dentist can be soldered in one minute, and in a manner superior to any other mode, without being placed in connection with any combustible matter. The work when finished, has the appearance of being cast in a mould.

The furnace is so constructed, that the heat does not affect the external part of the machine, after producing and keeping up for hours the most intense heat that is required by the metallurgist, the varnish on the external part of the instrument is not injured. When the blast from the bellows is not required, the heat may be kept up for any length of time, upon the principle of the air furnace; and it is so arranged, that the fuel can be kept ignited for hours, without consumption.

The instrument combines beauty with utility, and there is no piece of philosophical apparatus more ornamental.

R. SOMERBY.

*Description of Engraving.—a.* A perspective view of Dr. R. Somerby's concentrated blow-pipe and furnace; *b.* the lamp; *c.* lamp-stand; *d.* blow-pipe; *e.* cock, to cut off the air from blow-pipe; *f.* slide to raise or lower the lamp; *g.* top of table; *h.* cover to the furnace; *i.* the furnace; *j.* the pan to receive the ashes from the furnace; *k.* the valve at bottom of furnace; *l.* the pipe leading from the bellows to the furnace; *m.* the stop-cock, to cut off the wind from furnace; *n.* the main pipe leading from the bellows to the furnace and blow-pipe; *o.* the bellows; *p.* weight on the top of bellows; *r.* the treadle; *s.* the table legs.

## Figures and Tables

**Figure f1:**